# Structural and optical properties of a radio frequency magnetron-sputtered ZnO thin film with different growth angles

**DOI:** 10.1186/1556-276X-7-55

**Published:** 2012-01-05

**Authors:** Ki-Han Ko, Yeun-Ho Joung, Won Seok Choi, Mungi Park, Jaehyung Lee, Hyun-Suk Hwang

**Affiliations:** 1 School of Electrical, Electronics and Control Engineering, Hanbat National University, Daejeon, 305-719, Republic of Korea; 2 LG Display Co., Ltd., 1007 Deogeun-Ri, Wollong-Myeon, Paju, 413-811, Republic of Korea; 3 School of Information and Computer Engineering, Sungkyunkwan University, Suwon, 440-746, Republic of Korea; 4 Department of Electrical Engineering, Seoil University, Seoul, 131-702, Republic of Korea

**Keywords:** ZnO film, growth angle, antireflection coating, RF magnetron sputtering, solar cell

## Abstract

This study introduces optical properties of a columnar structured zinc oxide [ZnO] antireflection coating for solar cells. We obtained ZnO films of columnar structure on glass substrates using a specially designed radio frequency magnetron sputtering system with different growth angles. Field-emission scanning electron microscopy was utilized to check the growth angles of the ZnO films which were controlled at 0°, 15°, and 30°. The film thickness was fixed at 100 nm to get a constant experiment condition. Grain sizes of the ZnO films were measured by X-ray diffraction. A UV-visible spectrometer was used to measure the transmittance and reflectance of the ZnO film columnar structures as a function of the growth angles.

## Introduction

To achieve a high efficient solar cell, one of the most important processes is antireflection coating [ARC] which also has a function of passivation [1]. ARCs generally reduce the reflection of sunlight and increase the intensity of radiation on the inside of solar cells. With the antireflection layer, Choi et al. [2] demonstrated that solar cell efficiency can be increased by around 10%.

In general, the refractive index of a thin film is variable according to the kind of material and thickness of the films. It is addressed that a medium refractive index material between air (*n *= 1) and Si (*n *≈ 3.4) is optimal for the ARC [1]. However, with glass-based solar cells, such as dye-sensitized and thin film solar cells, it is hard to get a good antireflection effect due to a low refractive index of the glass substrate (*n *≈ 1.7). Therefore, with the glass base, a structural modification of the ARC is a better approach than the refraction effect scheme.

ZnO thin films are used in various applications due to their high optical transmittance in the visible light region [3]. ZnO, one of the most important binary II-VI semiconductor compounds, has a hexagonal structure and a natural n-type electrical conductivity [4]. Moreover, ZnO thin films doped with Al, Ga, or In have low electrical resistivity and high optical transmittance due to their high carrier concentrations above 10^20 ^cm^-3 ^and wide optical bandgap energy above 3.3 eV. Also, it has merits on having a low material cost, on being nontoxic, and on having a better stability under hydrogen plasma compared with ITO [5].

In this paper, we introduce the optical properties of columnar structured ZnO films formed with several different growth angles. The films were deposited with radio frequency [RF] magnetron sputtering. During the sputtering, the angle between the sample and the target (ZnO) is changed to get several different growth angles. Field-emission scanning electron microscopy [FE-SEM] was applied to check the growth angles of ZnO films, controlled at 0°, 15°, and 30°, and to measure the thickness of the film. The film thickness was fixed at 100 nm to get the same mechanical condition of the columnar structured thin films. The grain sizes of the ZnO films were obtained by X-ray diffraction [XRD]. A UV-visible [UV-vis] spectrometer was used to measure the transmittance and reflectance of the columnar structured ZnO films, as a function of the growth angles.

## Experiments

**Figure 1:**
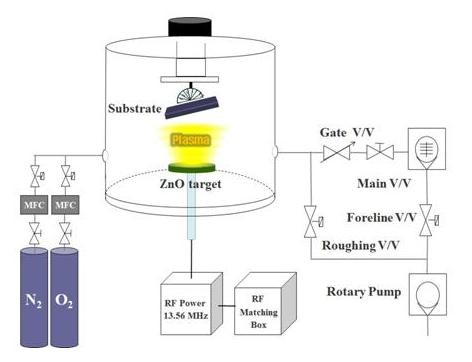
**A schematic of the RF magnetron sputtering system**.

Figure 1 shows a schematic of the film deposition apparatus to achieve different growth angles of the ZnO films. A 99.99% ZnO target was fixed, and a sample holder was mechanically tilted to get several different angles against the target. With many experiments and SEM measurements, we could get several different growth-angled ZnO films. The ZnO thin films were deposited on a glass (OA-_10_G, Nippon Electronic Glass Co., Ltd., Otsu, Shiga, Japan) substrate using the described RF magnetron sputtering system.

To get good quality samples, the glass substrates were cleaned in trichloroethylene, acetone, methanol, and distilled water for 10 min, respectively. The sputtering chamber was vacuumed to a base pressure of 1 × 10^-5 ^Torr. A pre-sputtering treatment was performed to clean the target surface for 10 min using argon plasma. A distance between the target center and the sample substrate was kept at 9.5 cm, and we manually tilted the sample substrate with angle measurement. The thickness and cross-sectional images of the films were measured by FE-SEM (Hitachi, S-4800, Hitachi High-Tech, Minato-ku, Japan); the grain sizes of the films were measured using XRD (Max 2500H, Rigaku Corporation, Tokyo, Japan), and the optical properties were observed in a UV-vis spectrometer (S-3100, Scinco, Gangnam-gu, Seoul, South Korea).

## Results and discussion

**Figure 2:**
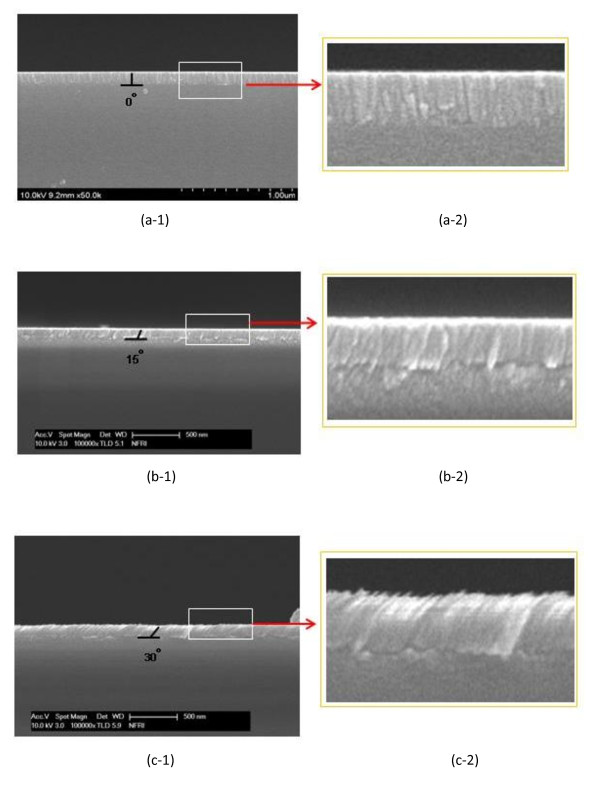
**FE-SEM images of ZnO films with various growth angles**. ZnO films at (**a-1**) 0°, (**b-1**) 15°, and (**c-1**) 30° growth angles and their enlarged images (**a-2**, **b-2**, and **c-2**).

Figure 2 shows cross-sectional FE-SEM images of the ZnO films with three growth angles. Film thickness is of the same value (100 nm). Figure 2a-1 shows the cross-section view of the 0° growth-angled columnar ZnO film. Columnar ZnO films with angles of 15° and 30° are shown in Figure 2b-1, c-1. To get a magnified view of the cross-section of the films, we enlarged the boxed section of the films, as shown in Figure 2a-2,b-2,c-2. The FE-SEM images confirm that the columnar ZnO films were successfully formed on glass substrates with different growth angles.

**Figure 3:**
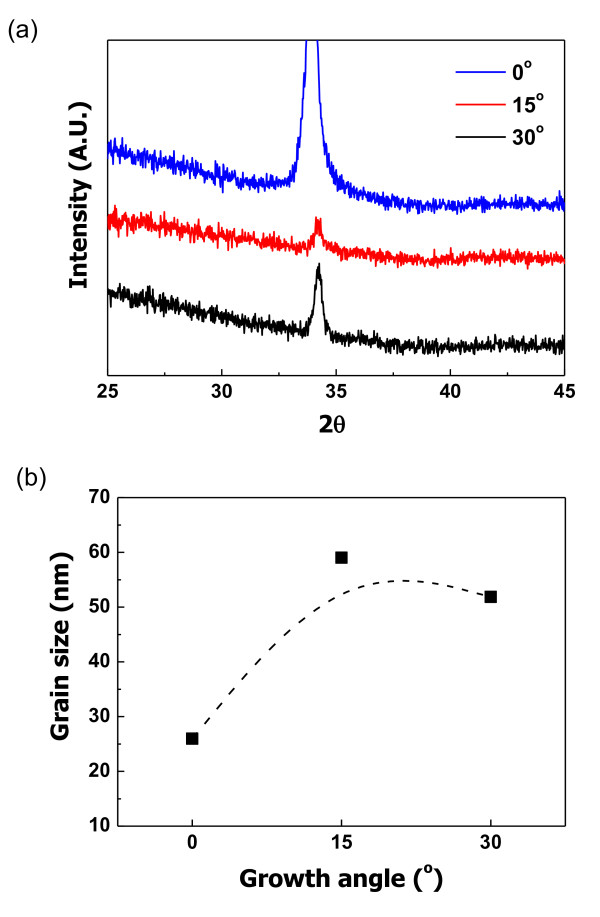
**XRD patterns of ZnO films vs. growth angles**. (**a**) X-ray spectra and (**b**) grain sizes.

Figure 3 shows the XRD patterns and grain sizes of the ZnO films according to growth angles. Figure 3a shows that all ZnO films had orientation peaks. The intensities of the main peaks are different according to growth angles. The highest peak is observed at the 0° angled columnar ZnO film. Figure 3b shows the grain sizes of the ZnO films according to growth angles. The biggest grain size is obtained at the 15° angled columnar ZnO film at 59.01 nm. The 0° growth-angled film has the smallest grain size at 25.95 nm. The grain size was calculated by getting the full width at half maximum value, according to Scherrer's equation [6].

**Figure 4:**
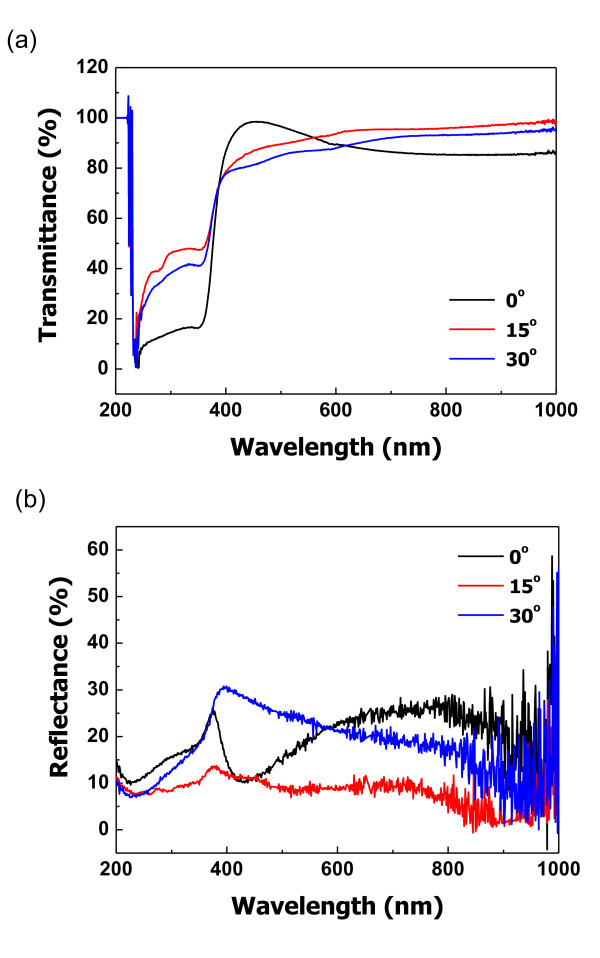
**Optical properties of ZnO films based on growth angles**. (**a**) Transmittance spectra and (**b**) reflectance spectra.

Figure 4a shows the transmittance patterns of ZnO films with different growth angles. All ZnO films show high transmittance above 90%. The The 0° angled columnar film has the highest transmittance, and the value is approximately 99% at 450 to approximately 500 nm. Figure 4b shows the reflectance patterns of the ZnO films. All ZnO films showed different patterns according to the wavelength of incidence rays. The ZnO film with a 0° growth angle has the lowest reflectance of 10.81% at 418 nm. The 15° angled film has the best condition on average and low values. The transmittance and reflectance are slightly changed by the growth angle of ZnO films.

**Figure 5:**
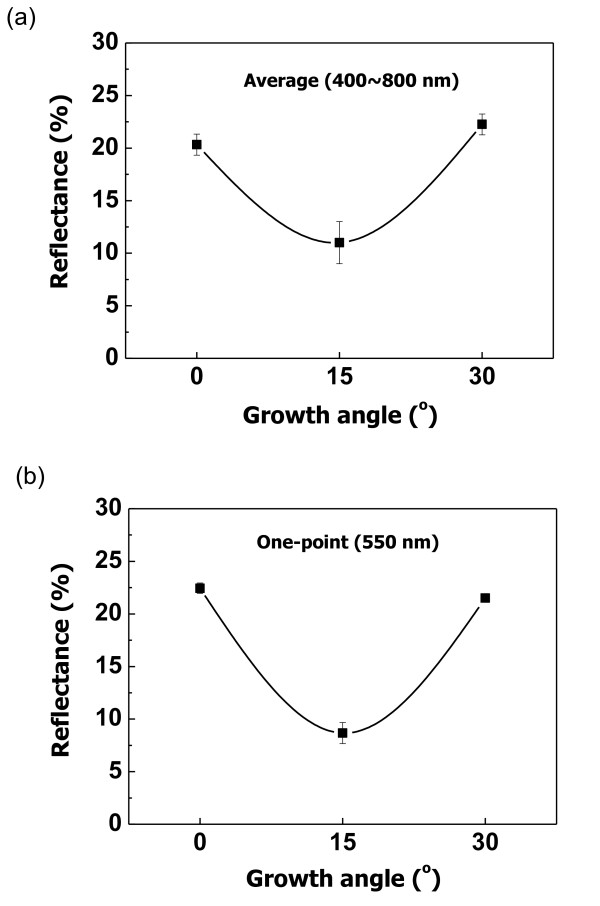
**Reflectance of ZnO films vs. growth angles**. (**a**) Average spectra and (**b**) one-point spectra.

Figure 5 shows the reflectance patterns of the ZnO films with different growth angles. Figure 5a shows the average reflectance in the wavelength range of 400 to 800 nm, and Figure 5b shows the reflectance at 550 nm wavelength. The 15° angled columnar ZnO film has the lowest average reflectance of 11%. Also, the reflectance at 550 nm wavelength is the lowest value at 8.67%. In addition, the lowest reflectance occurred when the reflected rays on the ZnO film got to a 15° angle. However, the 0° and 30° angle reflectances tended to increase compared with that of the 15° angle reflectance.

## Conclusions

We investigated the optical properties of antireflection coating on columnar structured ZnO films. The ZnO films were deposited on glass substrates inside a specially designed RF magnetron sputtering system. We studied the growth angle effect of the films for optical properties. The thickness of the ZnO thin films was checked by FE-SEM and was fixed at 100 nm. Three growth angles (0°, 15°, and 30°) of the columnar ZnO films were carefully selected. The intensities of the main peaks were different according to the growth angles. The highest intensity was obtained at the 0° angled columnar structured ZnO film. The 15° angled columnar structured film had the largest grain size of 59.01 nm, and the 0° angled columnar structured film had the lowest grain size of 25.95 nm. These results showed that intensity and grain sizes varied according to the growth angles. Transmittance of the ZnO thin films was changed according to the wavelength of incidence rays and the growth angle. The lowest average reflectance at 550 nm was measured with the 15° angled columnar thin film with a value of 8.67%. The best optical properties of the columnar structured ZnO films were obtained from the 15° angled growth columnar thin film.

## Competing interests

The authors declare that they have no competing interests.

## Authors' contributions

Y-HJ and WSC participated in the sequence alignment and drafted the manuscript. K-HK and MP carried out the sample preparation. JL and H-SH performed data acquisitions and analysis. All authors read and approved the final manuscript.
